# Residual cancer cells after apparent complete pathological response to neoadjuvant therapy in oesophageal adenocarcinoma

**DOI:** 10.1093/bjs/znae103

**Published:** 2024-04-17

**Authors:** Robert C Walker, Jack Harrington, Stella P Breininger, Oliver Pickering, Samuel L Hill, Benjamin P Sharpe, Ben Grace, Ian Reddin, Rushda Rajak, Antigoni Manousopoulou, Spiros D Garbis, Zoë S Walters, Matthew J J Rose-Zerilli, Timothy J Underwood

**Affiliations:** School of Cancer Sciences, Faculty of Medicine, University of Southampton, Southampton, UK; School of Cancer Sciences, Faculty of Medicine, University of Southampton, Southampton, UK; School of Cancer Sciences, Faculty of Medicine, University of Southampton, Southampton, UK; School of Cancer Sciences, Faculty of Medicine, University of Southampton, Southampton, UK; School of Cancer Sciences, Faculty of Medicine, University of Southampton, Southampton, UK; School of Cancer Sciences, Faculty of Medicine, University of Southampton, Southampton, UK; School of Cancer Sciences, Faculty of Medicine, University of Southampton, Southampton, UK; School of Cancer Sciences, Faculty of Medicine, University of Southampton, Southampton, UK; Pathology, University Hospital Southampton NHS Foundation Trust, Southampton, UK; Proteome Exploration Laboratory, Beckman Institute, California Institute of Technology, Pasedena, California, USA; Proteome Exploration Laboratory, Beckman Institute, California Institute of Technology, Pasedena, California, USA; School of Cancer Sciences, Faculty of Medicine, University of Southampton, Southampton, UK; School of Cancer Sciences, Faculty of Medicine, University of Southampton, Southampton, UK; School of Cancer Sciences, Faculty of Medicine, University of Southampton, Southampton, UK

## Introduction

Surgery, with or without neoadjuvant chemotherapy (nCT) or neoadjuvant chemoradiotherapy (nCRT), is the definitive treatment for patients with oesophageal adenocarcinoma (OAC)^1,2^. Multimodal treatment results in long-term changes to quality of life, raising interest in the possibility of omitting surgery for patients who achieve a cCR to neoadjuvant therapy^3^. It is not known whether it is safe to omit surgery, or the best ways of defining a cCR^4^.

This question is being addressed in several studies, including the SANO trial, in which bite-on-bite biopsies taken 6 and 12 weeks after completion of nCRT are being used to guide treatment decision-making. The preliminary results suggest non-inferiority of active surveillance, although questions remain about the decision to delay surgery in patients with a cCR, especially given the high incidence of distant cancer recurrence in the surveillance arm, and the validity of the tissue sampling method used to detect viable residual disease^5^.

The present study assessed whether residual cancer could be detected in the scar tissue at the primary site of OAC that had a pCR to neoadjuvant therapy. Conventional pathological approaches were supplemented with expanded immunohistochemistry, bulk RNA and proteomics assessment, and single-cell RNA (scRNA) sequencing.

## Methods

Resection and biopsy specimens were sampled directly in the operating theatre using an 8-mm punch biopsy, with normal oesophageal tissue being taken from the proximal resection margin. Pathological staging was performed in accordance with AJCC guidelines, and tumour regression classified according to the Mandard tumour regression grade (TRG)^6^. Proteomic and bulk RNA sequencing was performed on snap-frozen tissue samples, and scRNA sequencing was completed on disaggregated fresh samples using the DropSeq technique, as described previously^7–10^. Full methods are detailed in the *Supplementary material*.

## Results

### Conventional pathology and bulk sequencing failed to identify residual cancer cells

A cohort (cohort 1) of five patients undergoing oesophagectomy was selected, two of whom were treatment-naive, two had progressive disease following nCT, and one achieved a pCR (TRG1 in the primary tumour with no viable tumour on inspection of the tumour bed and resected lymph nodes) to nCT after routine pathological assessment (surgery 4–6 weeks after nCT).

**Fig. 1:**
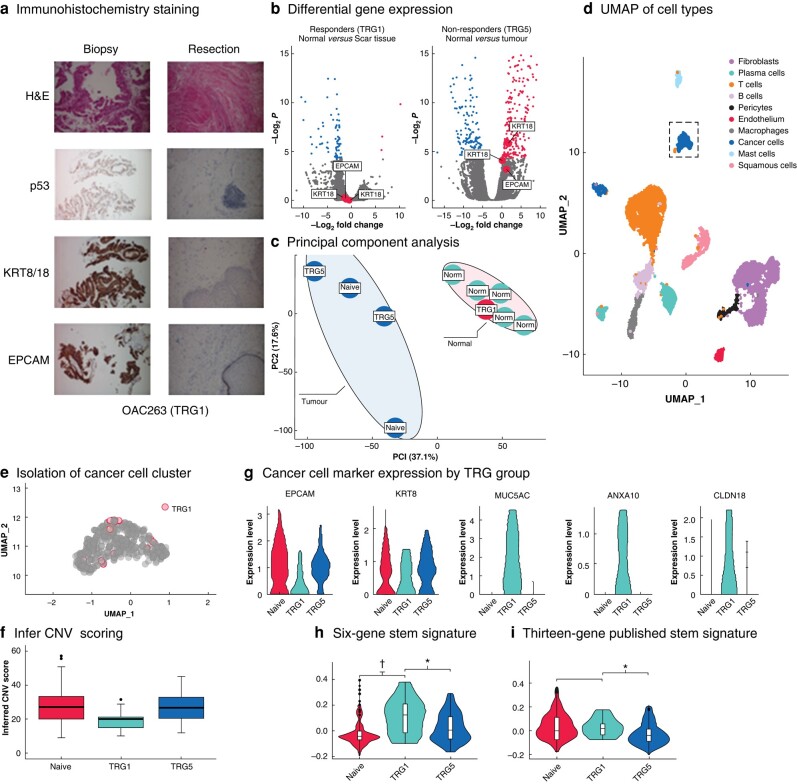
Assessment of cohort 1 using immunohistochemistry, bulk RNA sequencing, and shotgun proteomics together with analysis of Dropseq data that revealed a population of 12 cancer cells originating from the patient with a pCR to neoadjuvant chemotherapy **a** Haematoxylin and esoin (H&E) staining showing poorly differentiated adenocarcinoma in the biopsy sample but absence of cancer cells in the resection specimen. TP53 antibody stained the nuclei of cancer cells in the biopsy, and cytokeratin 8/18 and EPCAM antibodies highlighted the cancer cells in the biopsy. These cells were absent from the resection specimen. **b** Volcano plots from differential gene expression analysis of bulk RNA sequencing data between tumour and normal tissue in (left) responders and (right) non-responders. **c** Principal component analysis of proteomic data between tumour and matched normal samples. The tumour samples with no response (tumour regression grade (TRG) 5) or no exposure (naive) to neoadjuvant therapy demonstrated heterogeneity, whereas the normal oesophageal tissue samples formed a tight cluster alongside the tumour sample from the complete responder (TRG1). **d** Uniform manifold approximation and projection for dimension reduction (UMAP) projection single-cell RNA sequencing data highlighting cell types from five patients’ samples (cohort 1). **e** Isolation of cancer cell cluster showing cancer cells captured from a TRG1 complete responder to chemotherapy. **f** InferCNV scoring displaying the chromosomal abnormalities of the cancer cells including cells isolated from patients with TRG1. The increased score from 0 (normal) demonstrated a greater CNV in the cancer cells in relation to the normal cell populations indicative of malignancy. Median values (bold line) i.q.r. (box), and range (error bars) with outliers (dots) are shown. **g** Expression of cancer cell markers *EPCAM* and *KRT8* and the oncogenic genes *MUC5AC, ANXA10*, and *CLDN18* in the cancer cells of each sample (in TRG groups). **h** Module score assigned with the expression of *ALDH1A1, SOX9, ITGA6, SOX2, EZH2, and ALDH2*. **i** Module score of the expression of *ALDH1A1, ALCAM, CD24, CD44, PROM1, LGR5, EPCAM, MYC, MSI1, NANOG, DPP4, FUT4, ITGB1, BMI1, SALL4, KLF4, SOX2, and OCT4* showing enrichment of expression in post-treatment samples from TRG1 compared with treatment-naive or non-responders (TRG5). *P < 0.050, †*P* < 0.001 (Wilcox test).

Representative tissue from the tumour bed demonstrating a pCR, along with diagnostic pretreatment biopsies underwent additional staining for p53, KRT8/18, and EPCAM, to further assess for cancer cells. No evidence of cancer could be found in the resection specimen (*Fig. 1a*).

Bulk RNA profiling was undertaken, including the scar at the site of tumour regression in the TRG1 sample. CK8 (*KRT8*), CK18 (*KRT18*), and *EPCAM* were expressed differentially (upregulated) in TRG5 tumours, but undetectable in the regressed lesion (*Fig. 1b*).

Global shotgun proteomic profiling was completed on all samples, resulting in profiling of 10 657 proteins (peptide false discovery rate less than 0.05), with progressing tumours demonstrating a distinct profile compared with normal tissue, whereas the TRG1 samples aligned closely with normal tissue (*Fig. 1c*).

### Single-cell RNA sequencing

The cellular landscape of OAC was determined using scRNA sequencing (26 patients, including 28 tumour and 16 normal tissue samples)^10^. After data quality control, a total of 42 388 cells were available for assessment. These were classified into 10 cell lineages based on canonical marker gene expression and *a priori* knowledge (*Fig. S1a–c*). To confirm the identity of the 4452 malignant cells identified from tumours by their expression of OAC-associated genes (*EPCAM*, *AGR2*, *CEACAM6*, *KRT8*, *KRT18*, and *KRT19*), acquired copy number alterations were characterised using InferCNV. Reclustering of these malignant cells revealed 27 distinct clusters, which (over 75% cells per cluster) grouped based on the patient of origin^10^ (*Fig. S1d*,*e*).

The scRNA sequencing data from the patients in cohort 1 revealed two broad lineages for the cancer cells, one dominated by the expression of *SPINK4* and *CD24* composed mostly of cancer cells from a single patient, and the other containing cells from several patients and dominated by expression of *SLPI*, *KRT18*, and *KRT7* (*Fig. 1d*). Within this second cluster were 456 cancer cells, including 12 cells from the scar site of the patient treated with chemotherapy who achieved a pCR (*Fig. 1e*). These 12 cells expressed *EPCAM* and *KRT8*, as well as *MUC5AC*, a marker of Barrett’s oesophagus, and *ANXA10*, which promotes growth in oesophageal squamous cell carcinomas^11^ (*Fig. 1g*).

Identification by scRNA sequencing of residual cancer cells not observed by bulk approaches or current clinical gold standard histopathological assessment could represent the detection of a rare but clinically significant population. This observation in a single patient, who had received nCT as opposed to nCRT, which is favoured by trials in organ-sparing approaches, prompted further investigation in a second cohort (cohort 2).

### Matched pre–post-treatment sample assessment

Paired samples from three patients were included in cohort 2 for assessment. This included two patients treated with nCRT, one of whom experienced a pCR (TRG1 in the primary tumour) and one with progressive disease (TRG4) (surgery 8–10 weeks after nCRT). The third patient elected not to receive neoadjuvant treatment, but had tissue taken for scRNA sequencing at the time of diagnosis and then later at definitive surgery.

**Fig. 2:**
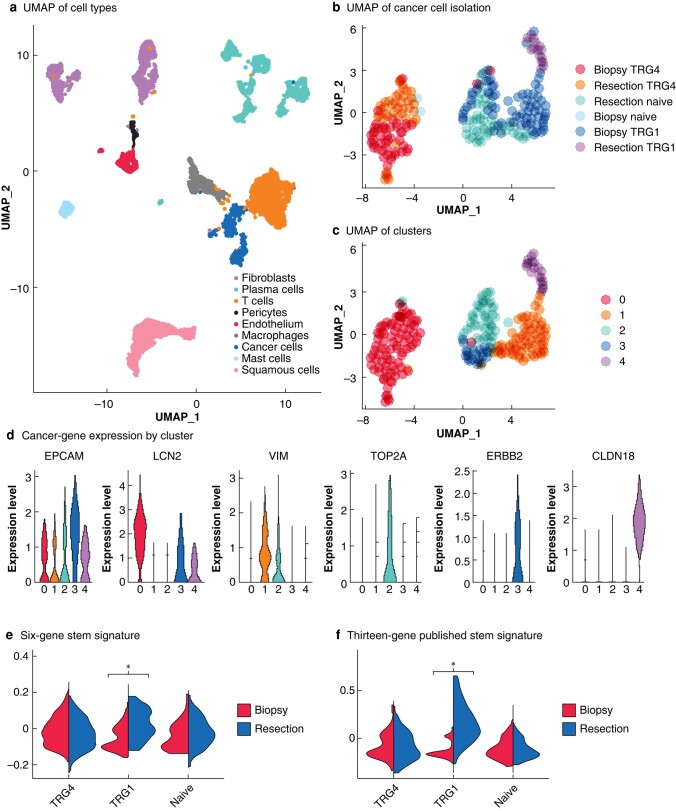
Analysis of dropseq data revealing a population of cancer cells originating from the patient with a pCR to neoadjuvant chemoradiotherapy **a** Uniform manifold approximation and projection for dimension reduction (UMAP) displaying cell types identified in second cohort. **b**,**c** UMAP plots demonstrating the isolation and reclustering of cancer cells. This led to the identification of five subpopulations. Notably, cluster 4 was predominantly composed of tumour regression grade (TRG) 1 residual cancer cells and clustered separately from cancer cells from the same patient before treatment. Pre- and post-treatment cancer cells from the patient with a TRG4 response also clustered distinctly. **d** Differentially expressed genes between each cancer cell subpopulation; cluster 4 was marked with raised expression of CLDN18. **e** module score assigned with the expression of *ALDH1A1, SOX9, ITGA6, SOX2, EZH2, and ALDH2* showing enrichment of expression in post-treatment samples from TRG1 compared with treatment naive or non-responders (TRG4). **f** Module score of expression of *ALDH1A1, ALCAM, CD24, CD44, PROM1, LGR5, EPCAM, MYC, MSI1, NANOG, DPP4, FUT4, ITGB1, BMI1, SALL4, KLF4, SOX2, and OCT4*. *P < 0.001 (Wilcox test).

In total, 6410 high-quality cells clustered into the same broad cell lineages as described previously (*Fig. 2a*). The cancer cells from cohort 2 were reclustered into five populations, which included a population of 45 cancer cells identified from the scar of the patient with a pCR after nCRT (*Fig. 2b*,*c*). These cancer cells had survived nCRT and clustered independently (*Fig. 2c*, cluster 4) of the pretreatment cancer cells from the same patient. They were marked by increased expression of claudin 18 along with other markers of a cancer stem cell phenotype (*Fig. 2d*).

### Residual cells expressed cancer stem cell markers

Genes expressed differentially between residual cancer cells and matched pretreatment biopsy specimens from the patient with a pCR (TRG1) treated with nCRT included the known cancer stem cell markers *SOX2* and *ALDH1A1*^12,13^. To interrogate the potential that these rare residual cancer cells represented OAC cancer stem-like cells, a 6-gene panel of cancer stem cell markers from the differentially expressed gene list was generated and used alongside a recently published 13-gene panel of cancer stem cell markers^14^. Both gene sets were significantly enriched in the two TRG1 post-treatment samples, but not the treatment-naive or TRG4 samples (*Figs 1h*,*i* and *2e*,*f*).

## Discussion

Using scRNA sequencing, rare populations of residual cancer cells within the scar tissue of primary tumours labelled as showing a pCR after neoadjuvant treatment were identified. That these cancer cell populations were not detectable by routine approaches, expanded immunohistochemistry, bulk RNA and proteomic assessment, points to their rarity, although their significance is unknown. This is pertinent given the current interest in organ-sparing approaches in OAC. Clinical trials in this space have used routine immunohistochemistry of bite-on-bite biopsies taken at endoscopy, which may lack the tissue coverage and resolution needed to identify residual cancer, bringing into question the safety of this management approach.

These residual cancer cells exhibit a strong cancer stem cell phenotype, which might explain their inherent resistance to neoadjuvant therapy. These cells demonstrate upregulation of *CLDN18*; claudin proteins have an emerging role in regulation of cancer stem cell populations, and isoform CLDN18.2 is subject to ongoing investigation as a therapeutic target. Results from ongoing phase III studies are encouraging^15^ and it looks likely that CLDN-targeted therapeutics will soon become part of the treatment algorithm for advanced oesophageal cancer. Such observations could lead to attractive future adjuvant therapeutic approaches in the curative setting too.

## Supplementary Material

znae103_Supplementary_Data

## Data Availability

Primary data are available on request.
