# Supplementary material for: Residual cancer cells after apparent complete pathological response to neoadjuvant therapy in oesophageal adenocarcinoma
Source: Br J Surg. 2024 Apr 17;111(4):znae103. doi: 10.1093/bjs/znae103 (PMC11023542; doi:10.1093/bjs/znae103)
Supplement: znae103_Supplementary_Data [file znae103_supplementary_data.zip › Supplementary_Materials.docx]

**Residual cancer cells after apparent complete pathological response to neoadjuvant therapy in Oesophageal adenocarcinoma.**

Robert C. Walker ^1†^, Jack Harrington^1†^, Stella P. Breininger^1†^, Oliver Pickering^1^, Samuel L. Hill^1^, Benjamin P. Sharpe^1^, Ben Grace^1^, Ian Reddin^1^, Rushda Rajak^2^, Antigoni Manousopoulou^3^, Spiros D. Garbis^3^, Zoë S. Walters^1^, Matthew J. J. Rose-Zerilli^1^, Timothy J. Underwood^1^.

^1^ School of Cancer Sciences, Faculty of Medicine, University of Southampton, Southampton, UK

^2^ Pathology, University Hospital Southampton NHS Foundation Trust, Southampton, UK

^3^ Proteome Exploration Laboratory, Beckman Institute, California Institute of Technology, USA

^†^ Robert C. Walker, Stella P. Breininger and Jack Harrington contributed equally to this work.

^*^Correspondence: Timothy J. Underwood, Somers Cancer Research Building, Southampton General Hospital, Southampton, UK, SO16 6YD. Telephone: +44(0)2380777222. Email: [tju@soton.ac.uk](mailto:tju@soton.ac.uk) @timthesurgeon

**Supplementary Materials - Index**

| **Supplementary Methods** |  |
| --- | --- |
| Patients and sampling | *page 3* |
| Immunohistochemistry | *page 3* |
| Tissue quantitative proteomics analysis | *page 3* |
| Bulk RNA-sequencing  Single cell RNA-sequencing  Inferred copy number variation (InferCNV) analysis | *page 4*  *page 4*  *page 5* |
| **Supplementary Results** |  |
| Clinical journey of patients with residual cancer cells following apparent complete pathological response | *page 6* |
| **Supplementary Figures and Tables** |  |
| Figure S1 | *page 7* |
| Figure S2 | *page 7* |
| **References** | *page 8* |
|  |  |

**Supplementary Methods**

Patients and sampling

Resection and biopsy tissue samples were sampled directly in the operating theatre using an 8 mm biopsy punch. Normal esophageal tissue was taken from the proximal resection margin. Tissue samples for scRNA-seq were immediately placed in ice cold tissue storage solution (Miltenyi) and transferred to the laboratory. Tissue for bulk RNA extractions and proteomic profiling were snap frozen within the operating theatre and stored in the vapor phase of liquid nitrogen until use. Pathological tumor staging was performed in accordance with American Joint Committee on Cancer guidelines. Response to NAT was determined by a histopathologist and classified according to Mandard Tumor Regression Grading (TRG) system, on a scale from TRG1: complete regression (fibrosis without detectable tumor tissue) to TRG 5: tumor tissue without any evidence of regression(1).

Immunohistochemistry

Tissue sections were cut at 4 µm onto superfrost coated slides and were machine stained using the Dako Autostainer Link 48 according to manufacturer’s protocols. The tissue was stained with antibodies EPCAM (Dako; MSVA-326R, 1:50 dilution), CK 8/18 (Dako; IR094, ready-to-use), and p53 (Dako; IR616, ready-to-use). Stained slides were subjected to expert GI pathology review.

Tissue quantitative proteomics analysis

Samples were dissolved in 0.5 M triethylammonium bicarbonate, 0.05% sodium dodecyl sulphate. These were then subjected to pulsed probe sonication (Misonix, Farmingdale, NY, USA) and lysates were centrifuged (16,000 g, 10 min, 4°C). Supernatants were measured for protein content with infrared spectroscopy (Merck Millipore, Darmstadt, Germany). One hundred μg of protein was used per sample. Samples were then reduced, alkylated and enzymatically proteolyzed using trypsin. Peptides from each sample were labelled using the ten-plex TMT reagent kit (Thermo Fisher Scientific, Waltham, MA, US). Two ten-plex experiments were performed, each ten-plex with tissue biopsies (OAC and adjacent normal oesophagus) from five patients. Labelled peptides per ten-plex experiment were mixed and analyzed using two-dimensional liquid chromatography and tandem mass spectrometry as reported previously(2-4). A detailed description of proteomic data processing and analysis is presented in supplementary material.

Bulk RNA-sequencing

RNA was extracted from snap frozen chunks of tissue with a BioMasher (OMNI International), Qiashredder (Qiagen) and RNeasy Mini kit (Qiagen). Libraries were created using 500 ng of the extracted total RNA with a TruSeq® Stranded mRNA Library Prep kit (Illumina) before sequencing on the NextSeq 500 platform (Illumina) with 75 bp paired end reads. FASTQs were trimmed with Trimmomatic v0.32 before alignment to hg38 using STAR v2.7.1a with the quantMode argument to generate number of reads per gene for subsequent analysis in R.

Single cell RNA-sequencing

Patient tissues were disaggregated into single cell suspensions according to a modified protocol for fibroblast characterisation(5). Dropseq was performed following a slightly modified DropSeq protocol v3.1(6), where single cells from the suspension were co-encapsulated with a barcoded bead in nanoliter droplets. The droplets were broken and the bead yield underwent reverse transcription, followed by an exonuclease and PCR step. For each sample, 500 pg of cDNA were used for a Nextera XT library prep kit (Illumina) before sequencing on a NextSeq 500.  Raw reads were demultiplexed and converted to fastqs through bcl2fastq (Illumina). Fastqs were underwent the Dropseq Core Computational Protocol v2.1 pipeline, aligning with hg38 using STAR 2.6.0a. Subsequent analysis was performed with Seurat (R package version 4.1.1). The data was filtered for cell barcodes with 150 - 4,000 genes detected, less than 25% mitochondrial genes and less than 10% threshold for tissue dissociation signature genes(7). Counts were normalized and variable genes selected using SCTransform with latent variables to control for technical variation, prior to PCA, community detection (Louvain) and visualization using the RunUMAP() function. Cluster-specific genes were detected using a Wilcoxon rank sum test (p< 1 × 10^-3^ was considered significant). Cell identity was defined by the expression of canonical markers using *a priori* knowledge. After manual inspection of lineage markers of each dataset, further low-quality or doublet cells were removed, as well as patients with low cellularity (n=5). Module scoring was applied to the cancer cells using the MSigDB Hallmark collection(8, 9). Quiescent cells were not analyzed further due to the low number of cells present (n=256). For each separate cell lineage dataset, we iteratively pruned cells where we were uncertain of their cell identity (by marker log-normalized expression < 1). The pruned datasets were then re-clustered using the steps described above prior.

Inferred copy number variation (InferCNV) analysis

Copy number variation in malignant cells was performed using InferCNV(10-12). Samples retained for analysis were compared to a reference set of “normal” cells from the same samples using InferCNV with default settings except cutoff = 0.1, HMM = TRUE.

**Supplementary Results**

Clinical journey of patients with residual cancer cells following apparent complete pathological response

Given the complete pathological response observed within the primary tumor following nCT, it is expected that the prognosis would be good for the responding patient from the first cohort. Unfortunately, they experienced disease recurrence.

This patient, having proceeded to a laparoscopic Ivor-Lewis oesophagectomy following 3 cycles of neoadjuvant Oxaliplatin and Capecitabine chemotherapy given at full dose, recovered well in the immediate post operative period. While initial surveillance imaging and clinical reviews were reassuring, they eventually developed a clinically apparent mass within the right supraclavicular fossa, approximately 28 months after surgery. Cross-sectional imaging at this point revealed a diffuse metastatic pattern with pulmonary and mediastinal nodal relapse (Figure S2).

Progressive disease was observed after 6 months of palliative chemotherapy treatment at which point increasing lung, lymph node and new local anastomotic recurrence was apparent, and they were managed with best supportive care.

This patient’s journey contrasted with the individual from the second cohort whose TRG1 tumor was resected following nCRT. On completion of neoadjuvant therapy, they proceeded to surgery with a relatively uncomplicated admission. They recovered well, and entered routine surveillance, remaining without signs of relapse 3 years post-surgery.

**Supplementary Figures and Tables**

**Figure S1**

**Figure S1:** **The cellular ecosystem of EAC**. **A.** UMAP of cell lineages and their transcriptional complexity (violin plots). Identical UMAPs show lineage marker gene expression. **B.** Bar chart of each cell lineage by patient treatment status (***p<0.001). **C.** Cell type frequency between normal and tumor samples. **D.** UMAP of cancer cell populations. **E.** Pie charts for each cancer cell cluster in D, colored by patient ID. (Whole cohort, n=28)

**Figure S2**

**Figure S2**. **Comparison of response of primary gastro-esophageal junction (GOJ) tumor to neoadjuvant chemotherapy and pulmonary metastases to first line palliative chemotherapy.** **A**) Primary tumor at the GOJ prior to neoadjuvant therapy. **B.** Complete radiological regression of GOJ tumor following 4 cycles of neoadjuvant Oxaliplatin and Capecitabine. **C.** Pulmonary metastasis at time of systemic relapse more than 2 years after esophagogastrostomy. **D.** Good response of pulmonary metastasis to palliative Cisplatin, Capecitabine and Trastuzumab therapy, though without complete radiological response observed in the primary tumor.

**References**

1. Mandard AM, Dalibard F, Mandard JC, Marnay J, Henry-Amar M, Petiot JF, et al. Pathologic assessment of tumor regression after preoperative chemoradiotherapy of esophageal carcinoma. Clinicopathologic correlations. Cancer. 1994;73(11):2680-6.

2. Manousopoulou A, Woo J, Woelk CH, Johnston HE, Singhania A, Hawkes C, et al. Are you also what your mother eats? Distinct proteomic portrait as a result of maternal high-fat diet in the cerebral cortex of the adult mouse. Int J Obes (Lond). 2015;39(8):1325-8.

3. Manousopoulou A, Gatherer M, Smith C, Nicoll JAR, Woelk CH, Johnson M, et al. Systems proteomic analysis reveals that clusterin and tissue inhibitor of metalloproteinases 3 increase in leptomeningeal arteries affected by cerebral amyloid angiopathy. Neuropathol Appl Neurobiol. 2017;43(6):492-504.

4. Manousopoulou A, Koutmani Y, Karaliota S, Woelk CH, Manolakos ES, Karalis K, et al. Hypothalamus proteomics from mouse models with obesity and anorexia reveals therapeutic targets of appetite regulation. Nutr Diabetes. 2016;6(4):e204.

5. Waise S, Parker R, Rose-Zerilli MJJ, Layfield DM, Wood O, West J, et al. An Optimized Method to Isolate Human Fibroblasts from Tissue for ex vivo Analysis. Bio Protoc. 2019;9(23):e3440.

6. Macosko EZ, Basu A, Satija R, Nemesh J, Shekhar K, Goldman M, et al. Highly Parallel Genome-wide Expression Profiling of Individual Cells Using Nanoliter Droplets. Cell. 2015;161(5):1202-14.

7. van den Brink SC, Sage F, Vertesy A, Spanjaard B, Peterson-Maduro J, Baron CS, et al. Single-cell sequencing reveals dissociation-induced gene expression in tissue subpopulations. Nat Methods. 2017;14(10):935-6.

8. Liberzon A, Birger C, Thorvaldsdottir H, Ghandi M, Mesirov JP, Tamayo P. The Molecular Signatures Database (MSigDB) hallmark gene set collection. Cell Syst. 2015;1(6):417-25.

9. Subramanian A, Tamayo P, Mootha VK, Mukherjee S, Ebert BL, Gillette MA, et al. Gene set enrichment analysis: a knowledge-based approach for interpreting genome-wide expression profiles. Proc Natl Acad Sci U S A. 2005;102(43):15545-50.

10. Tirosh I, Venteicher AS, Hebert C, Escalante LE, Patel AP, Yizhak K, et al. Single-cell RNA-seq supports a developmental hierarchy in human oligodendroglioma. Nature. 2016;539(7628):309-+.

11. Tirosh I, Izar B, Prakadan SM, Wadsworth M, Treacy D, Trombetta JJ, et al. Dissecting the multicellular ecosystem of metastatic melanoma by single-cell RNA-seq. Science. 2016;352(6282):189-96.

12. Puram SV, Tirosh I, Parikh AS, Patel AP, Yizhak K, Gillespie S, et al. Single-Cell Transcriptomic Analysis of Primary and Metastatic Tumor Ecosystems in Head and Neck Cancer. Cell. 2017;171(7):1611-+.
