# Supplementary figures and images for: Residual cancer cells after apparent complete pathological response to neoadjuvant therapy in oesophageal adenocarcinoma
Source: Br J Surg. 2024 Apr 17;111(4):znae103. doi: 10.1093/bjs/znae103 (PMC11023542; doi:10.1093/bjs/znae103)

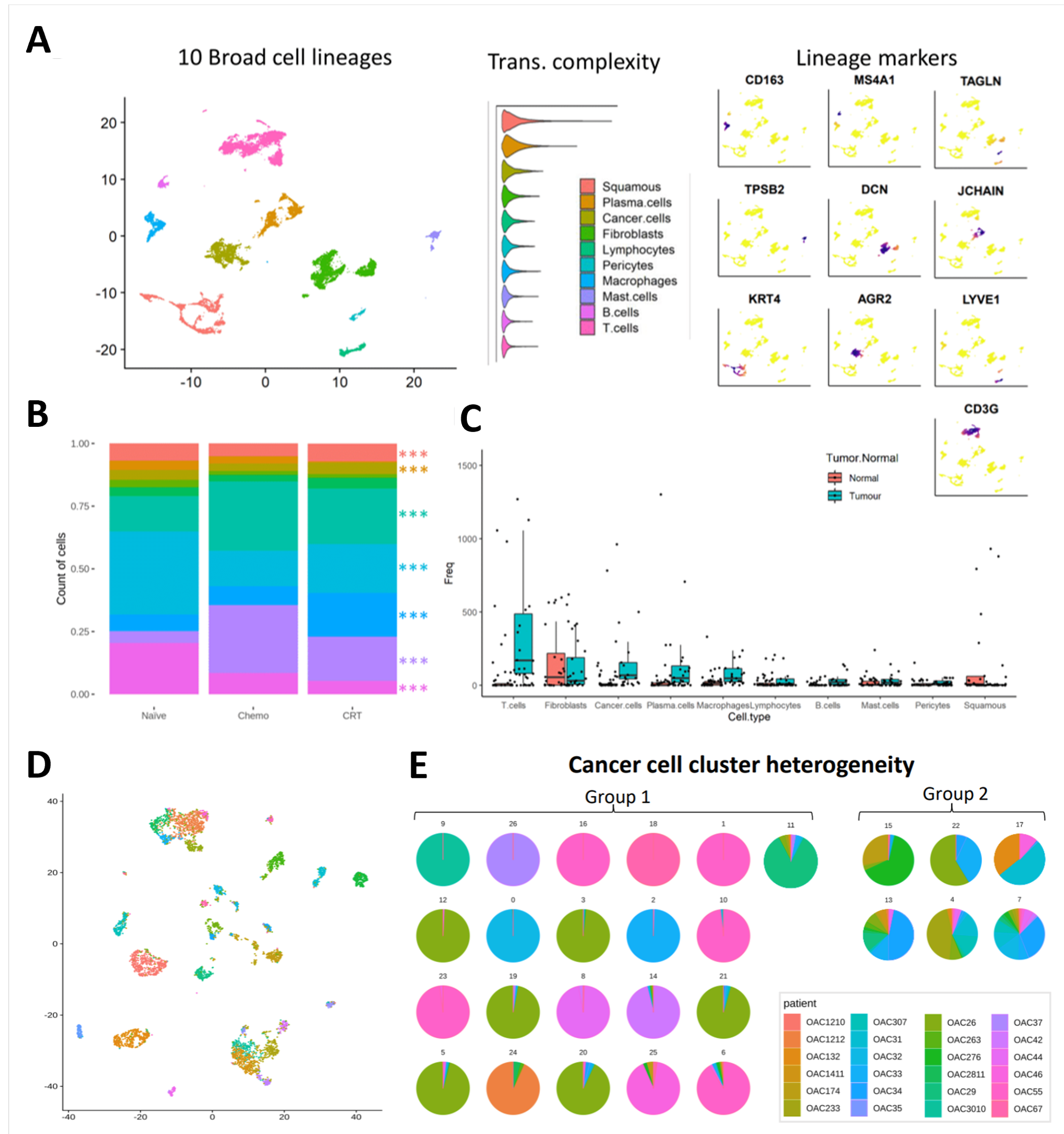

Supplement: znae103_Supplementary_Data [file znae103_supplementary_data.zip › Figure_S1.tiff]

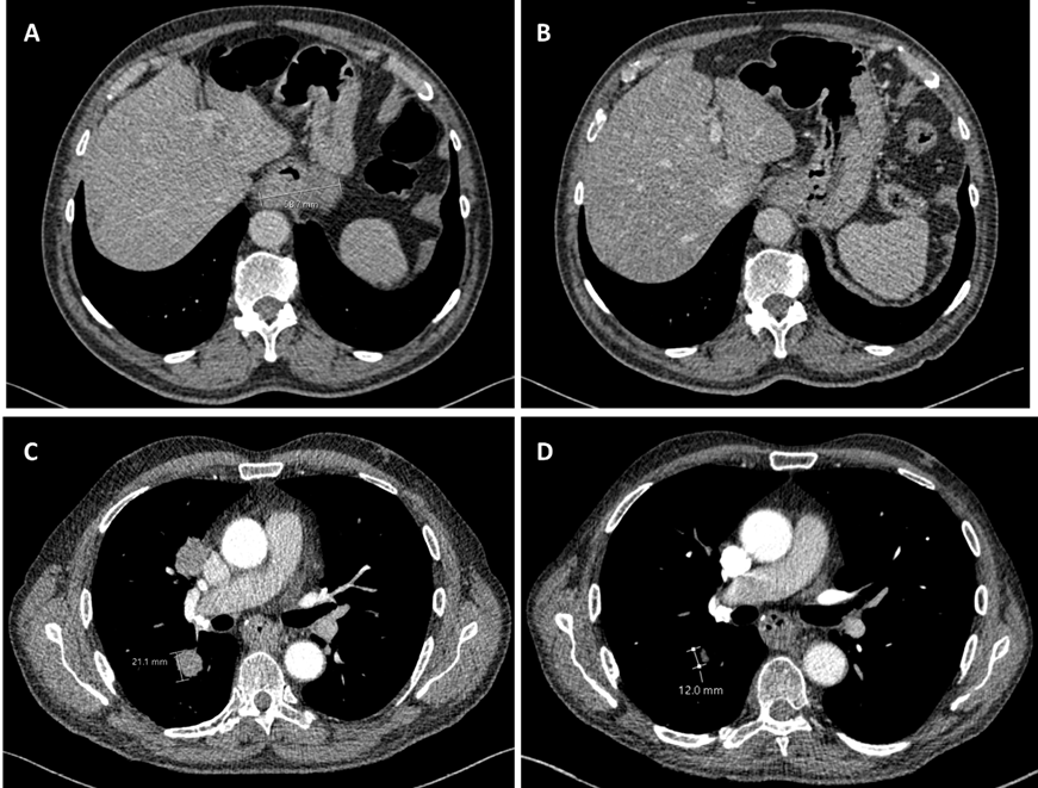

Supplement: znae103_Supplementary_Data [file znae103_supplementary_data.zip › Figure_S2.tiff]
